# Intersectional analysis of early childhood stunting in households in Nigeria

**DOI:** 10.1371/journal.pone.0355408

**Published:** 2026-08-20

**Authors:** Ayodeji Babatunde Oginni

**Affiliations:** Innovative Aid, Victoria, British Columbia, Canada; All India Institute of Medical Sciences - Raipur, INDIA

## Abstract

This study assessed the trend in the level of under-five stunting across the different dimensions of the intersection of household wealth status (rich, average, or poor), type of place of residence (rural or urban), and gender of household heads (male or female) to identify households that were persistently most at risk of under-five stunting in Nigeria. A secondary data analysis was conducted on nationally representative children’s data from four consecutive national surveys (Nigeria Demographic and Health Survey) conducted in 2003, 2008, 2013, and 2018 in Nigeria. The study outcome variable was under-five stunting status (stunted and “not stunted”). The primary independent variable was household type derived from the intersection of the gender of the household head, household wealth status, and type of place of residence. Meta-analyses with forest plots were used to determine log odds ratios of stunting in different household types using Rich Female-Headed Urban (RFHU) Households as the reference category. Multivariate analysis was also conducted using a Generalized Linear Model for each year’s dataset. The meta-analyses show that the odds of a child becoming stunted were significantly 4.26 times higher in PMHR, 3.25 times higher in PFHR, 2.92 times higher in PMHU, 2.59 times higher in AMHR, and 2.48 times higher in AMHU households compared to RFHU households. The multivariate models also show that from 2003, 2008, 2013–2018, PMHR (AOR: 5.12; 3.31; 4.22; & 3.72; p < 0.05), PMHU (AOR: 3.25; 2.75; 2.60; & 2.84; p < 0.05), PFHR (AOR: 3.58; 2.86; 3.47; & 3.07; p < 0.05), AMHU (AOR: 3.96; 2.47; 2.25; & 1.97; p < 0.05), and AMHR (AOR: 4.47; 2.31; 2.37; & 2.08; p < 0.05), consistently had significantly higher odds of predisposing under-five children to stunting than RFHU households. These findings offer fresh insights to guide policymakers in developing new policies and help program managers design and implement more tailored nutrition-sensitive interventions and programs for the households most at risk of under-five stunting in Nigeria.

## Introduction

Early childhood stunting (low height-for-age) is a nutritional outcome of chronic undernutrition in children under five and a measure of linear growth retardation and cumulative growth deficits, which reflect failure to receive adequate nutrition over a long period. Its most direct causes include inadequate nutrition (not eating enough or eating foods that lack growth-promoting nutrients) and recurrent infections or chronic diseases that cause poor nutrient intake and absorption [1].

**Fig 1:**
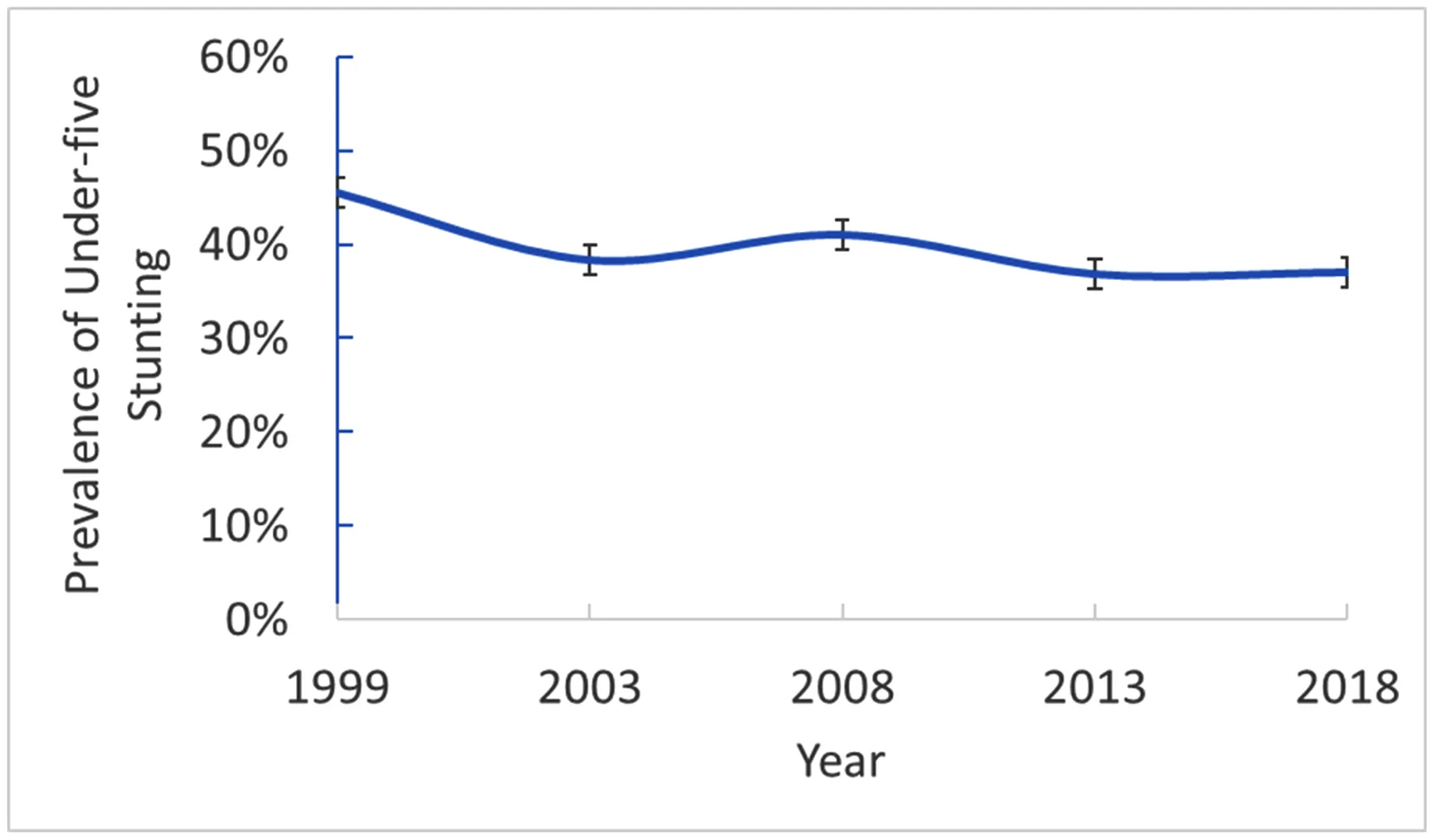
Trend in prevalence of under-five stunting in Nigeria, NDHS reports 1999-2018.

Early childhood stunting has early and long-term consequences from childhood to adulthood [2]. It increases the risk of developmental disorders (such as delayed language and gross motoric development), and poorer neuropsychological outcomes in children [3–5]. It is also associated with poor psychological functions in late adolescence. Stunted children are more likely to have anxiety, depressive symptoms, lower self-esteem, and poorer emotional and behavioral outcomes in late adolescence [6]. There is also a link between childhood stunting and economic outcomes, as a study reveals that increased stature is associated with increased wages for men and women [7]. Other consequences of stunting include increased morbidity (increased risk of infections and non-communicable diseases, increased susceptibility to fat accumulation mostly in the central region of the body, lower fat oxidation, lower energy expenditure, insulin resistance, and a higher risk of developing diabetes, hypertension, dyslipidemia, unfavorable maternal reproductive outcomes in adulthood), mortality, lowered working capacity, etc [2]. The physical and neurocognitive damage that accompanies stunted growth is potentially irreparable and thus constitutes the major obstacle to human development [2]. The economic consequence of stunting is equally huge, as it has been estimated that, on average, the GDP per Capita is 7% lower in Sub-Saharan Africa as a result of some of today’s workers being stunted in childhood [8]. The world has made progress to reduce child stunting, with a decrease in the prevalence from 26.4% in 2012 to 23.2% in 2024 [9]. However, in Africa, although the prevalence seemed to decrease from 34.0% in 2012 to 30.3% in 2024, the number of children who are stunted increased from 61.7 million to 64.8 million. In Nigeria, the prevalence of stunting remains very high despite the development and implementation of the National Strategic Plan of Action for Nutrition (2014–2025), including effective coordination of stakeholders and increased government commitment to funding for the procurement of ready-to-use therapeutic food, and the establishment of community-based management of acute malnutrition sites across the country, and other interventions [1,10,11]. According to the Nigeria Demographic and Health Survey Reports [1,12–15], the prevalence of under-five stunting in the country has dwindled from 46% in 1999 to 37% in 2018 (Fig. 1). Between 2012 and 2024, the prevalence of stunting reduced from 36.9% to 33.8%, but the number of affected children slightly reduced from 11.5 million to 11.4 million [9].

The probability of stunting in childhood substantially increases with risk accumulation in perinatal conditions, genetic factors, maternal conditions, and feeding practices; perinatal conditions are the main aspects associated with stunting [16]. Some specific household-level factors, such as location of residence, improved source of drinking water, household wealth index, household food insecurity, etc., and other individual-level variables such as maternal age, height, and educational level, history of not taking anti-parasite drugs during pregnancy, duration of breastfeeding, sex, and age of a child, have also been identified as the major drivers or predictors of children’s growth stunting [17–19]. Other common drivers include paternal education, sanitation conditions, maternal health services access, and family planning [20].Another important global concern is food price inflation, as it can exacerbate child malnutrition. From 2021 to 2023, food prices increased significantly more than prices for other consumer goods and services. This rise has placed an unfair burden on households that spend a large portion of their income on food, making it increasingly less affordable compared to other goods in the economy [9].

Despite the wealth of knowledge of the drivers of stunting in the literature, the gap in understanding that this study aimed to fill was to move beyond examining the effect of each driver or different aspects of a child’s identity to highlighting the quantitative effect of the interaction of two or more drivers on stunting. This intersectional approach to research is rooted in the theoretical framework that people who share one or more group identities can have different experiences, without presuming their experiences, and this approach critically examines differences in social class, status, and privilege that inevitably vary both within and between groups [21]. “*Drawing on black feminist and critical legal theory, Kimberlé Crenshaw developed the concept of intersectionality, a term she coined to speak to the multiple social forces, social identities, and ideological instruments through which power and disadvantage are expressed and legitimized.*” [22]. Quantitative nutrition research that incorporates intersectionality is gaining momentum, though it remains primarily focused on the United States and India. Additionally, none of these studies have explored indicators related to infant and young child nutritional outcomes [23].

Therefore, the objective of this study was to assess the trend in the level of under-five stunting across the different dimensions of the intersection of household wealth status, type of place of residence, and gender of household heads.

## Materials and methods

The study design was a secondary analysis of nationally representative children’s data from four consecutive national surveys (Nigeria Demographic and Health Survey) conducted in 2003, 2008, 2013, and 2018 in Nigeria. The survey samples were probability samples and the data were independent. The 2003 NDHS sample was selected using a two-stage cluster design; the 2008 NDHS sample was selected using a stratified two-stage cluster design; the 2013 NDHS sample was selected using a stratified three-stage cluster design; and the NDHS 2018 sample was selected using a two-stage stratified cluster design [1,13–15].

The outcome variable, “Stunting”, was derived from the variable “HW7” – “Ht/A Standard deviations”, which represents the standard deviations for height for age, according to the new WHO definition. Children whose height-for-age Z-score was below minus two standard deviations (−2 SD) from the median of the reference population were considered stunted. The outcome variable was binary (stunted and “not stunted”).

**Fig 2:**
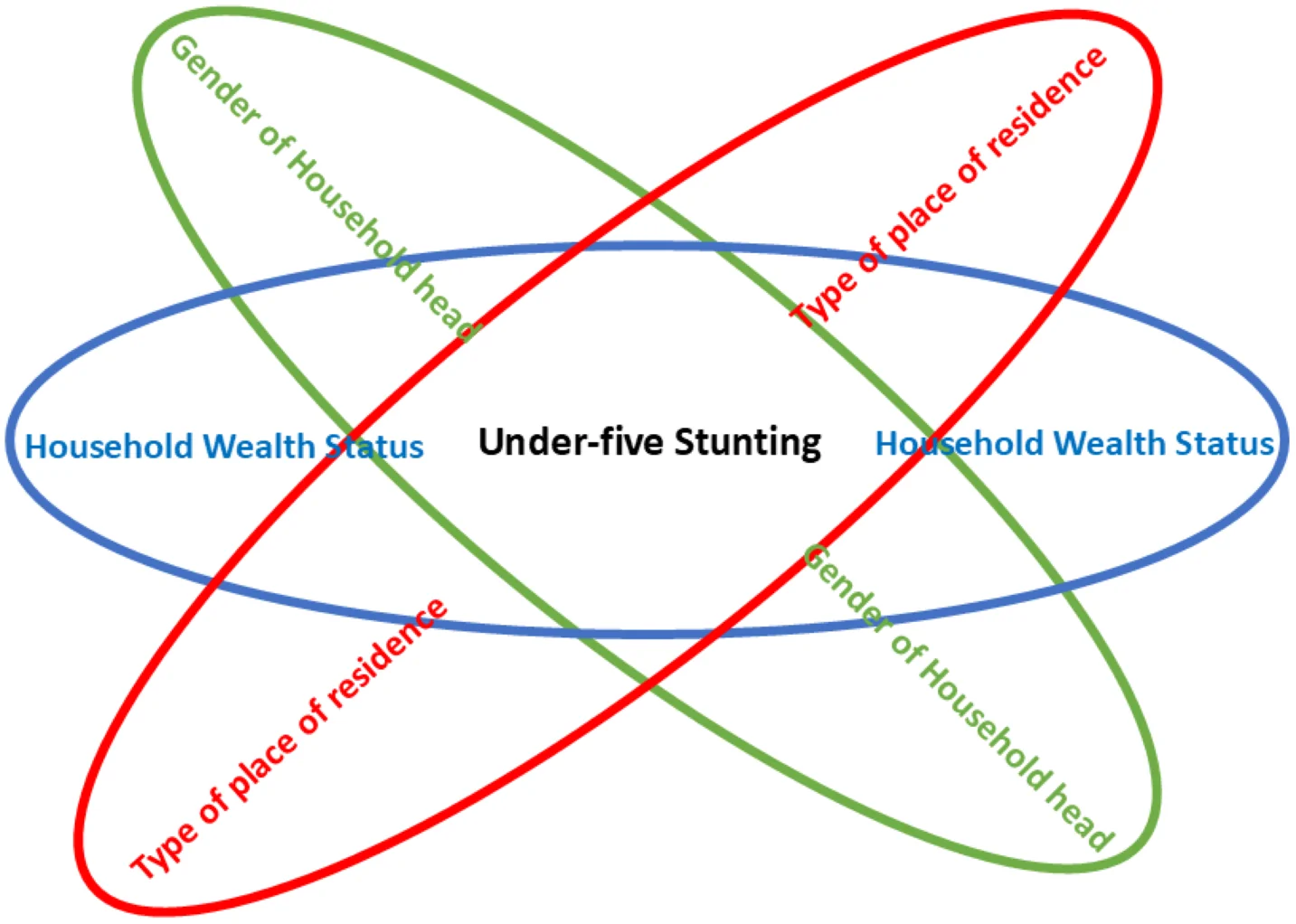
The conceptual framework for under-five stunting and the intersectionality of household wealth status, type of place of residence, and gender of household heads.

The conceptual framework for the study was rooted in the premise that under-five stunting is jointly shaped by multiple social positions of the household (e.g., Household wealth status, gender of household head, and type of place of residence), and cannot be adequately understood by considering social positions independently (Fig 2). The intersection of these multiple social positions creates intersectional variables such as different types of households with different experiences of under-five stunting. Most frequently, intersectional variables are included in regression models [24].

As shown in Table 1, the main independent variable was household type derived from the intersection of the gender of the household head (male-headed or female-headed), type of place of residence (rural or urban), and household wealth status (poor, average, or rich). The three variables used to create the main independent variable were “type of place of residence” (v102: 1-Urban, 2-Rural), “sex of household head” (v151: 1-male, 2-female), and “recoded wealth index (v190)”. The original variable “wealth index (v190)” has 6 categories (1-Poorest, 2-Poor, 3-Middle, 5-Rich, and 6-Richest), but it was recoded into a new variable with three categories (1-Poor, 2-Average, and 3-Rich). Combining the categories of these three variables gave 12 possible combinations, as shown in Table 1.

**Table 1:** Household types emanating from the intersection of household wealth status, gender of household head, and type of place of residence.

PMHU—Poor Male-Headed Urban Households	AMHU –Average Female-Headed Urban Households
PMHR –Poor Male-Headed Rural Households	AMHR – Average Female-Headed Rural Households
PFHU – Poor Female-Headed Urban Households	RMHU—Rich Male-Headed Urban Households
PFHR –Female-Headed Rural Households	RMHR –Rich Male-Headed Rural households
AMHU – Average Male-Headed Urban Households	RMHU –Rich Female-Headed Urban Households
AMHR – Average Male-Headed Rural Households	RMHR –Rich Female-Headed Rural Households

A supplementary dataset was created for the time series analysis of the under-five stunting estimates for each household type over the survey years. Supplementary datasets were also created for the Meta-Analysis Forest Plot, which was used to combine the log-odds ratios from the four surveys. The preference for the Meta-Analysis Forest Plot over pooled analysis was based on the need to visually display study-level results and heterogeneity.

A Generalized Linear Model (Binomial) was used to estimate the odds ratio (95% Confidence Intervals) of under-five stunting in each household type relative to the RFHU household for each year’s dataset, as shown in Table 3. Two models were developed for each year’s dataset, with “Stunting” as the outcome variable and household type as the primary independent variable (Household type). The first model (Model 1) comprised only the primary independent variable, while the second model (Model 2) incorporated the three control variables, which were “number of household members” (v136), “age of household head” (v152), and “number of children 5 and under” (v137). In total, four dependent variables were analyzed. The basis for selecting the included covariates is data availability and the theory of limiting the analysis to household-level variables.

The link function for the models is as follows:

Model 1: $\text{ln}\left(Odds(y=\text{1}\right))=\alpha +\beta_{\text{1}}x$

Model 2: $\text{ln}\left(Odds(y=\text{1}\right))=\alpha +\beta_{\text{1}}x+\beta_{\text{2}}x_{\text{2}}+\beta_{\text{2}}x_{\text{2}}+\beta_{\text{3}}x_{\text{3}}$

The models also accounted for the clustering effect of the variable “Region” having six clusters, and used the clustered robust standard error estimator. The variance inflation factor was estimated for Model 2 to check for multicollinearity. The goodness-of-fit test statistics (Deviance) comparing the fitted models to the saturated model (perfect fit) were reported in Table 2. Statistical significance was set at p < 0.05. The analyses were performed with StataNow/SE 19.5 for Windows (StataCorp LLC).

### Ethical considerations

This study involves the analysis of secondary data, utilizing publicly available and fully de-identified data. According to ethical guidelines from the U.S. Department of Health and Human Services (HHS) and the Common Rule (45 CFR 46), research utilizing publicly accessible datasets without identifiable personal information does not constitute human subjects research and does not require Institutional Review Board (IRB) approval [25].

No direct interaction with human participants occurred, and the data cannot be traced back to individuals. The dataset was obtained from the DHS Program, which ensures compliance with ethical and privacy standards. As no sensitive or confidential information is involved, this study poses no risk to individuals or communities. For these reasons, formal ethical approval was not required for this study.

## Results

**Fig 3:**
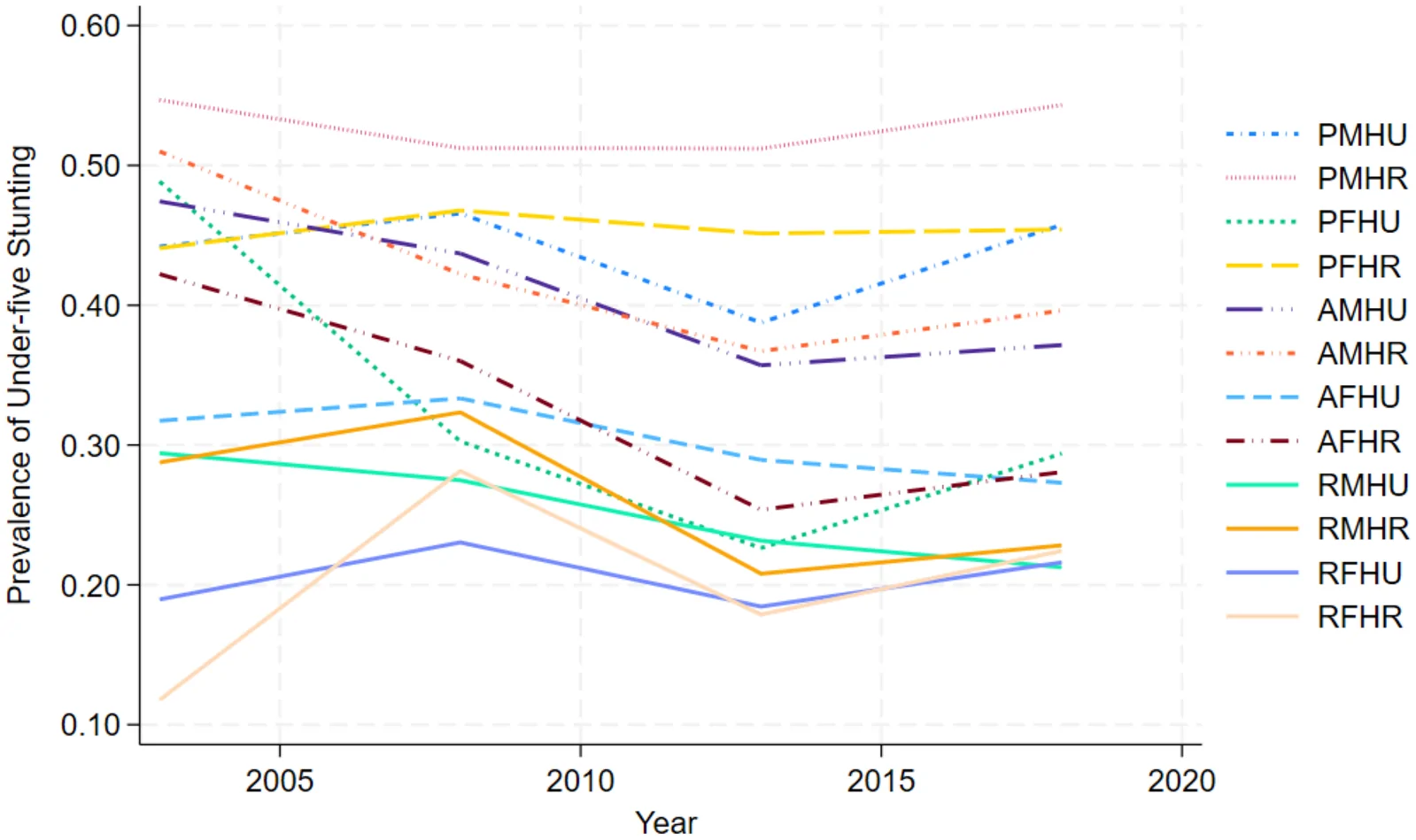
Trend in prevalence of under-five stunting by household type, 2003-2018.

Fig 3 presents the trend in the prevalence of under-five stunting across the 12 household types. PMHR households consistently had the highest prevalence of stunting, which stayed above the 50% mark from 2003 to 2018. The prevalence of under-five stunting in PFHR households stealthily increased over the period; meanwhile, the prevalence of under-five stunting in PFHU households reduced markedly over the same period to less than 30%. Although the prevalence of under-five stunting in PMHU households dwindled from 2003 to 2013, it increased from 2013 to 2018. However, the prevalence of under-five stunting in RFHR households, RFHU households, and RMHU households, though undulated from 2003 to 2018, never went above the 30%-point mark.

**Fig 4:**
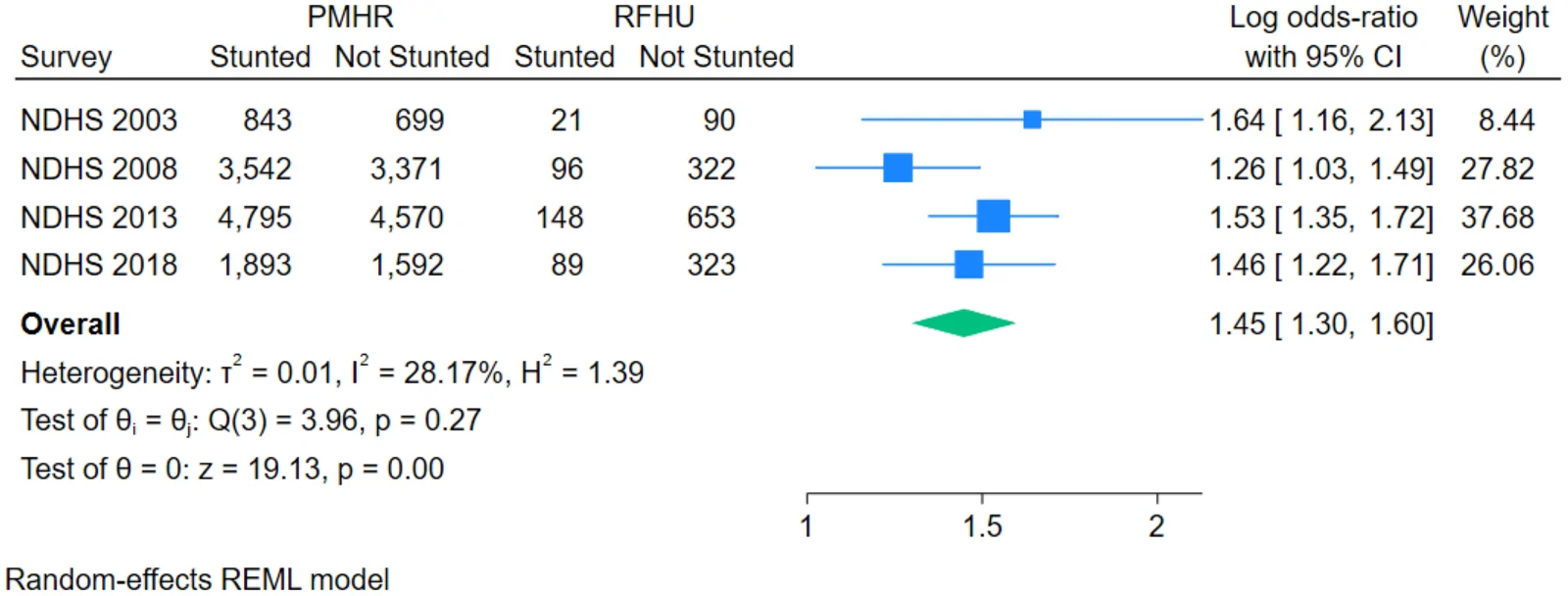
The odds of under-five stunting in PMHR versus RFHU.

**Fig 5:**
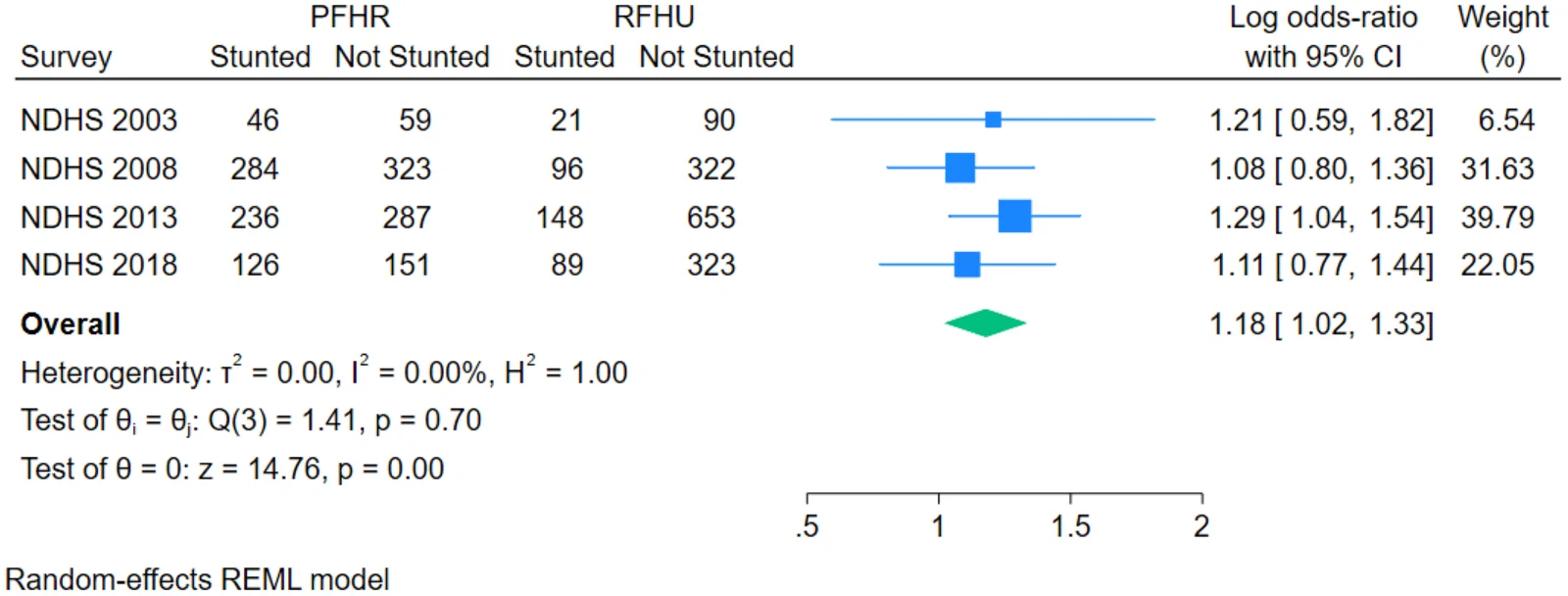
The odds of under-five stunting in PFHR versus RFHU.

**Fig 6:**
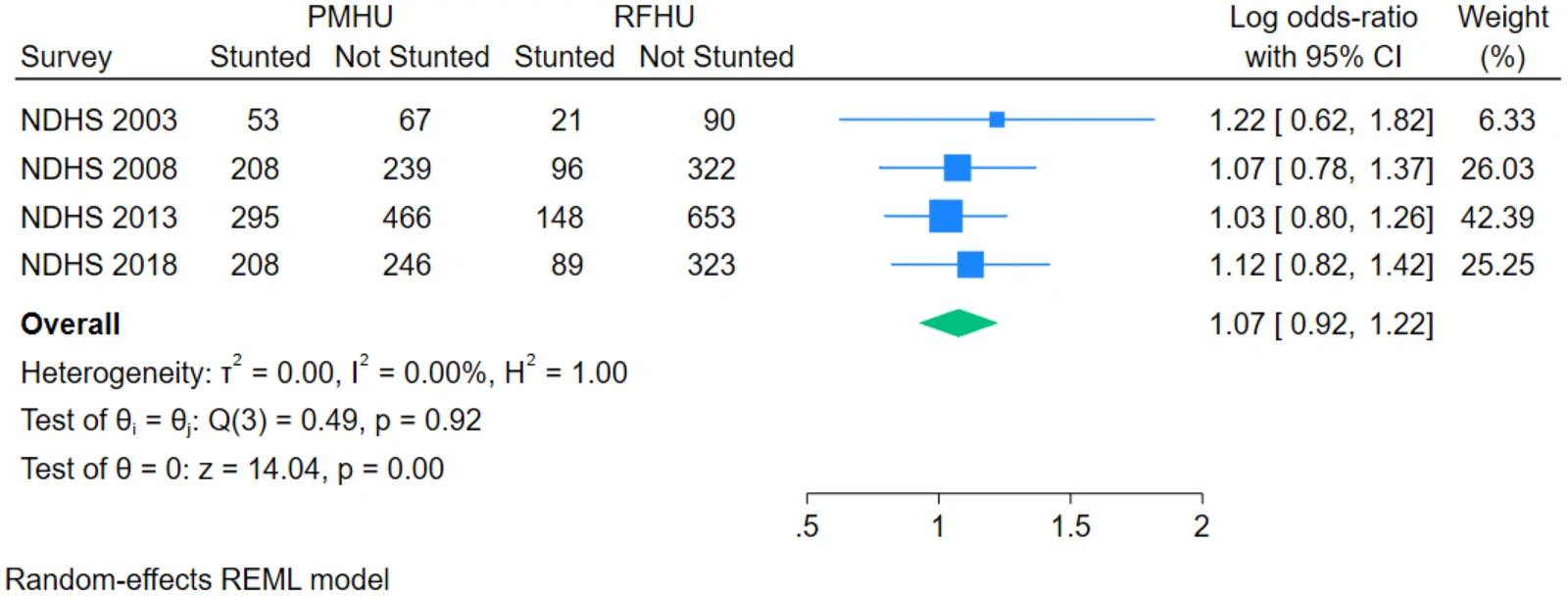
The odds of under-five stunting in PMHU versus RFHU.

**Fig 7:**
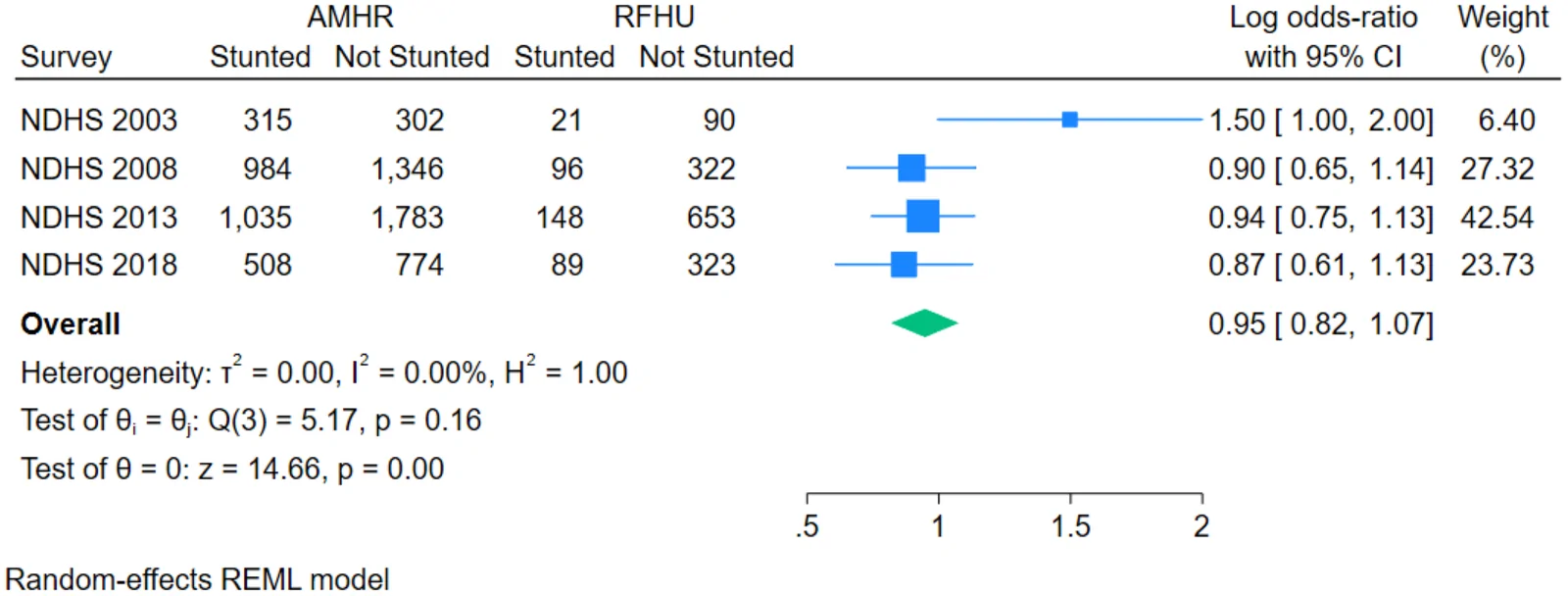
The odds of under-five stunting in AMHR versus RFHU.

**Fig 8:**
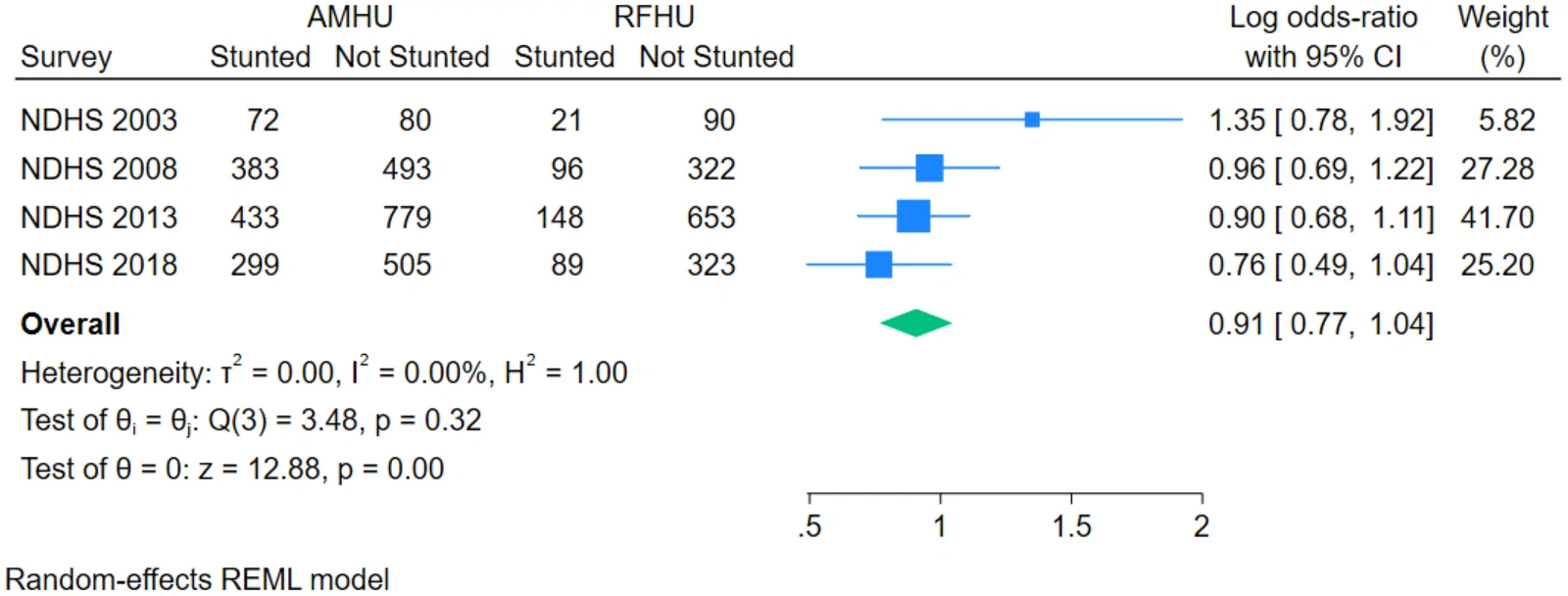
The odds of under-five stunting in AMHU versus RFHU.

**Fig 9:**
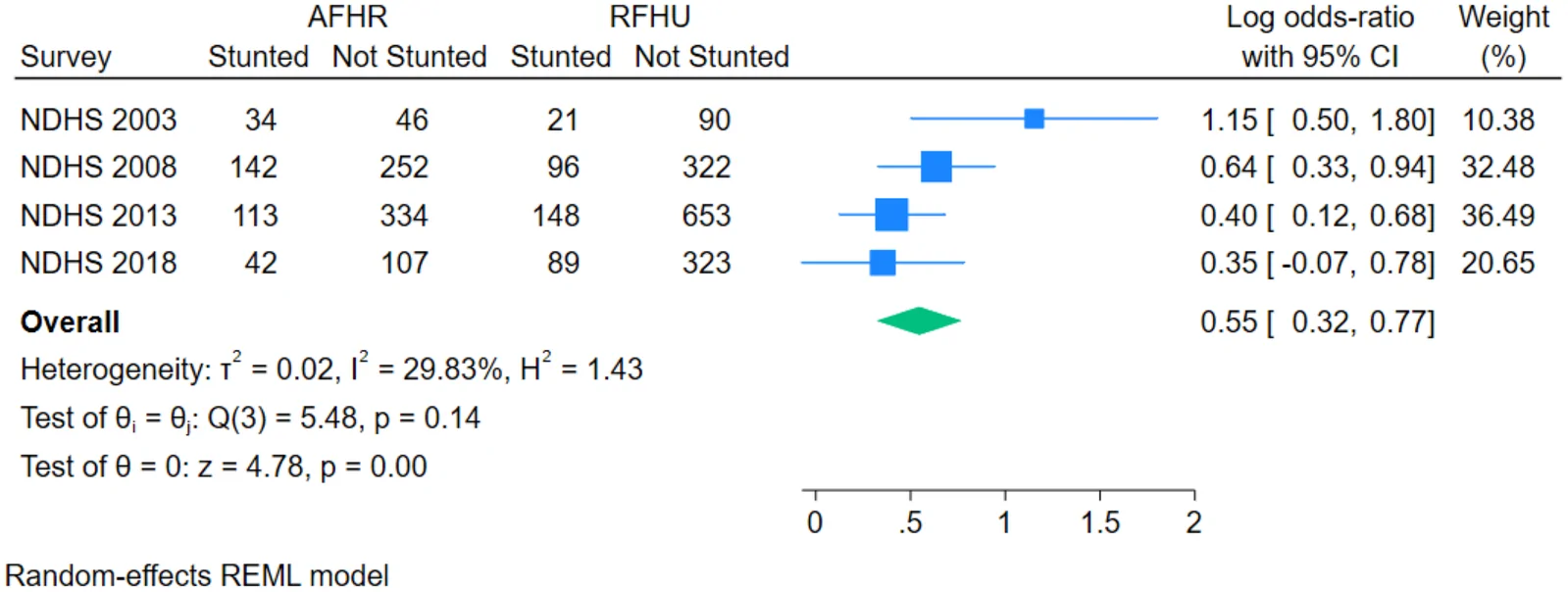
The odds of under-five stunting in AFHR versus RFHU.

**Fig 10:**
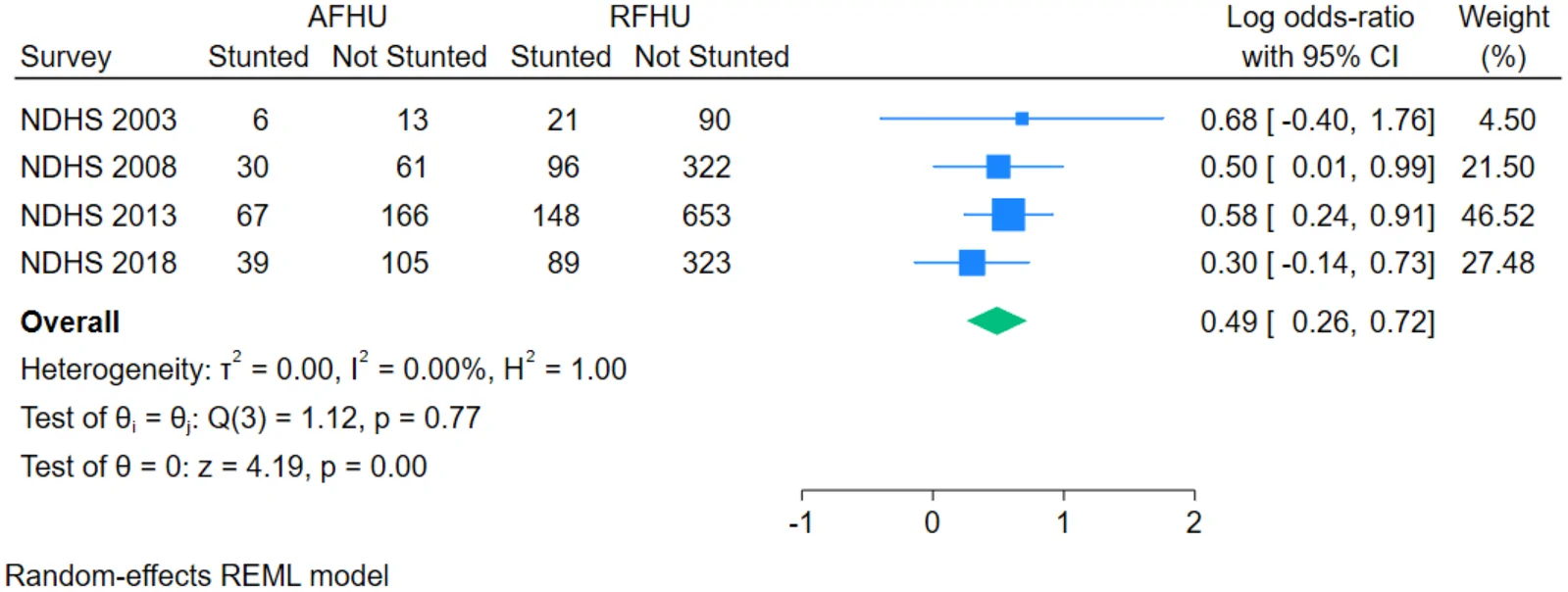
The odds of under-five stunting in AFHU versus RFHU.

**Fig 11:**
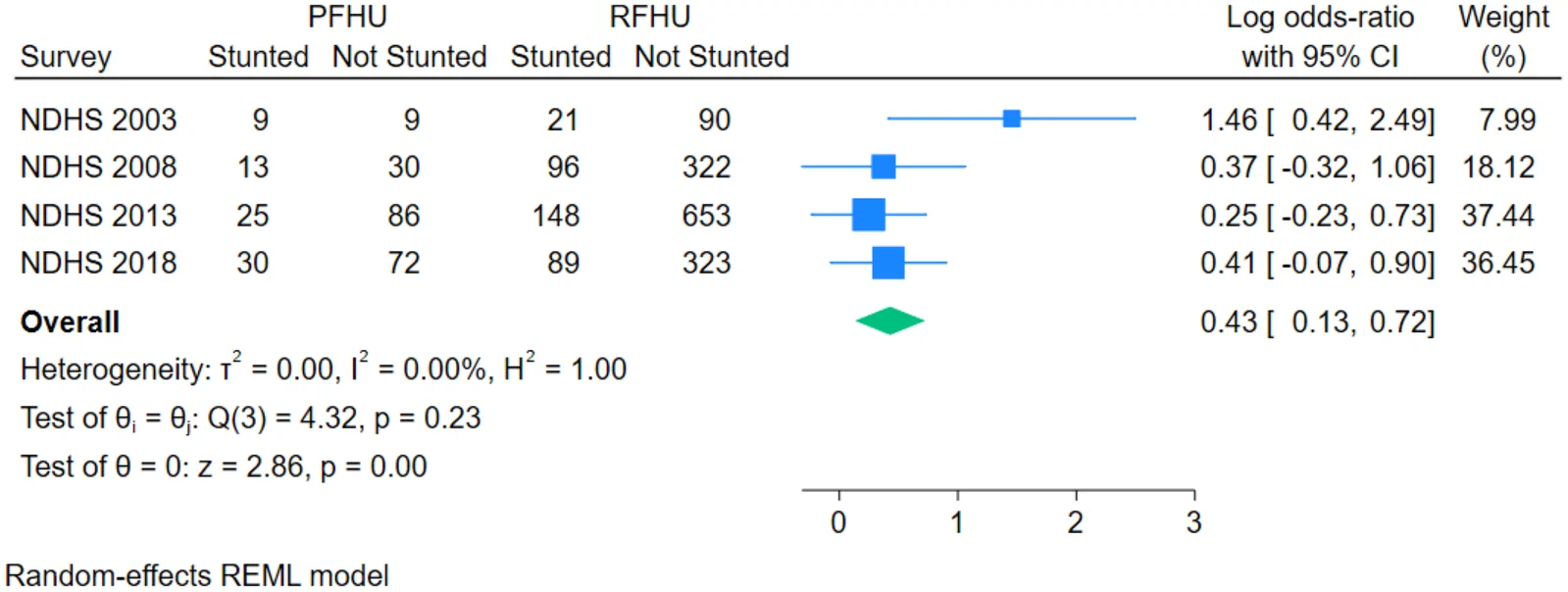
The odds of under-five stunting in PFHU versus RFHU.

**Fig 12:**
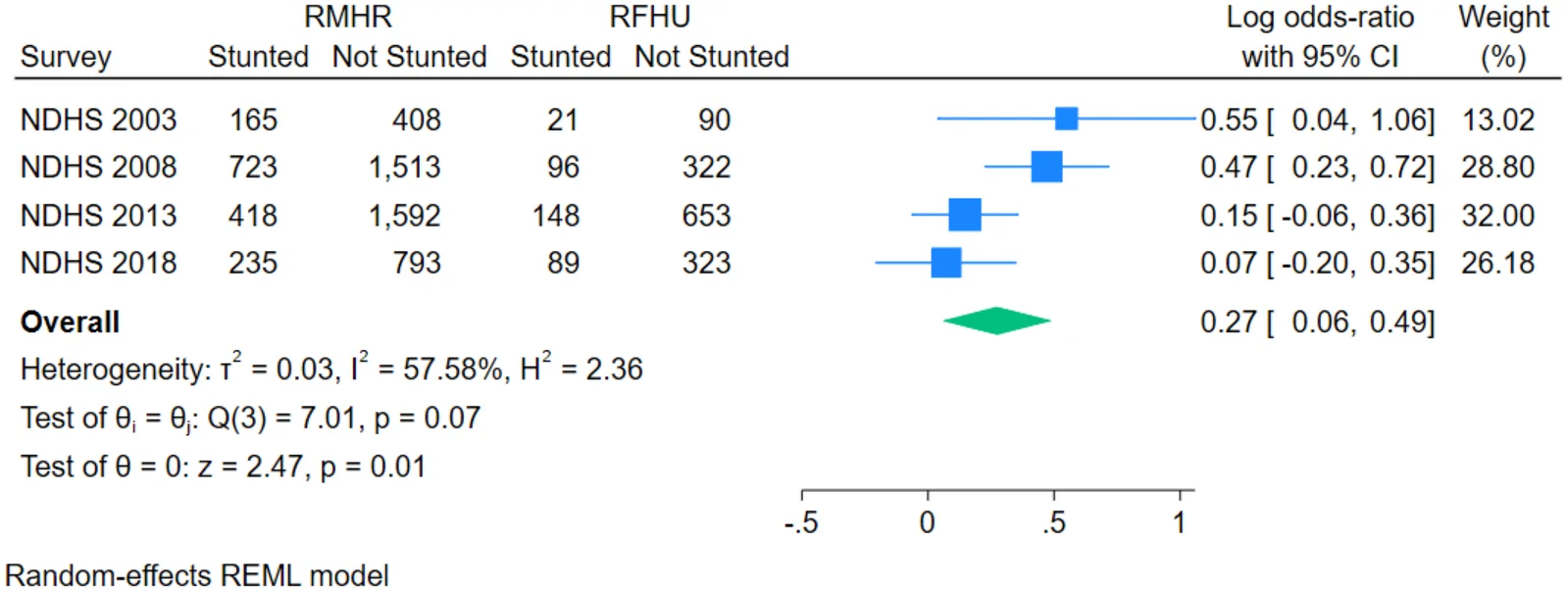
The odds of under-five stunting in RMHR versus RFHU.

**Fig 13:**
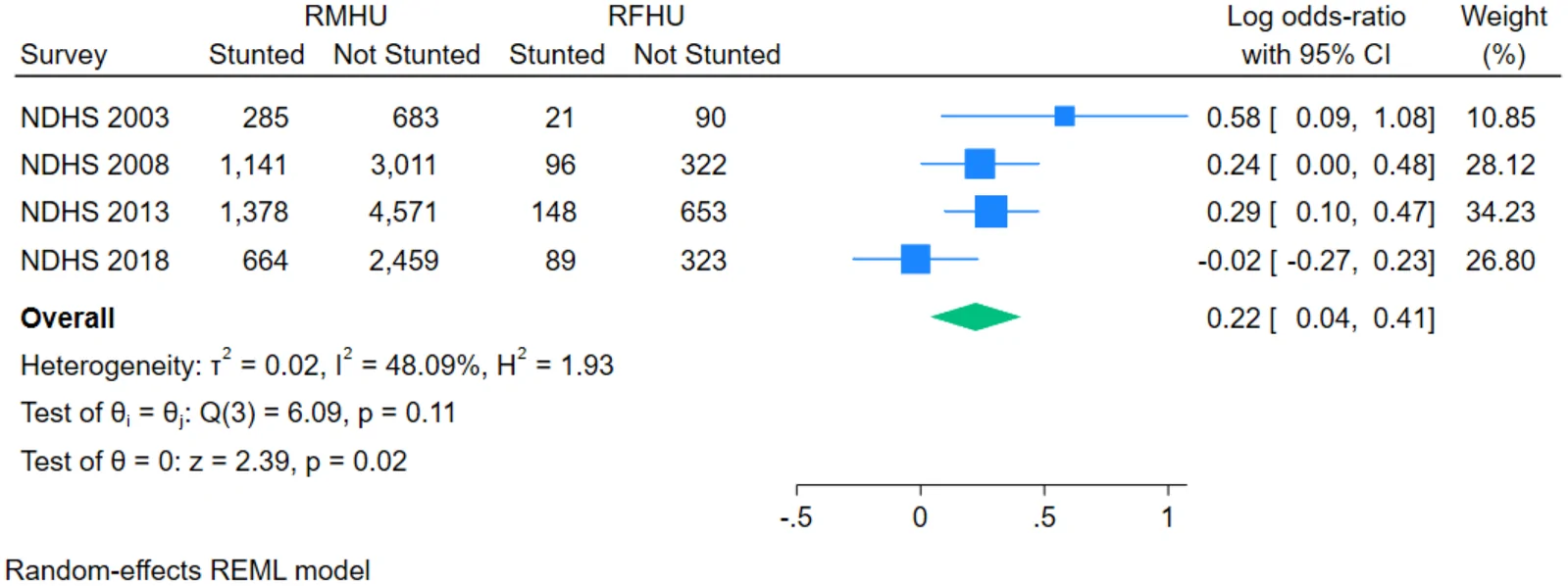
The odds of under-five stunting in RMHU versus RFHU.

**Fig 14:**
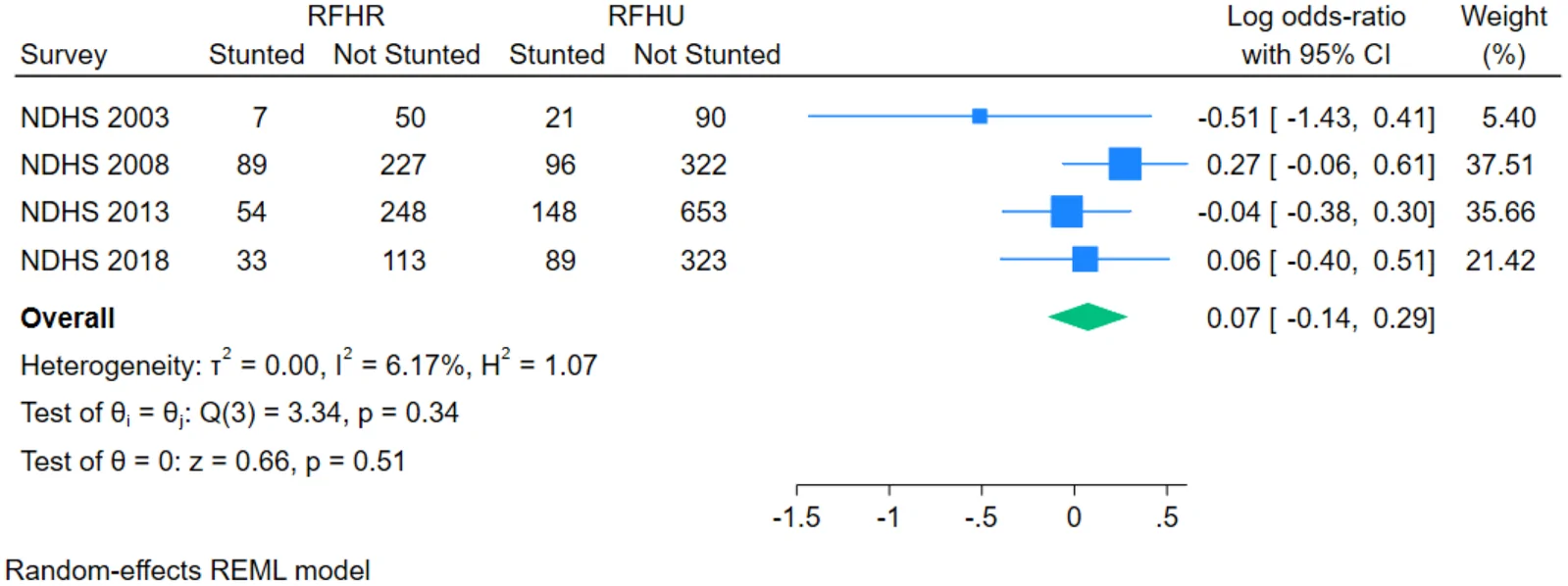
The odds of under-five stunting in RFHR versus RFHU.

Figs 4–14 present the summarized Log odds ratio estimates of under-five stunting in different household types compared to RFHU households, using forest plots. Among the household types, PMHR had the highest pooled estimate (Log OR: 1.45; 95% CI: 1.30–1.60), followed consecutively by PFHR (Log OR: 1.18; 95% CI: 1.02–1.33), PMHU (Log OR: 1.07; 95% CI: 0.92–1.22), AMHR (Log OR: 0.95; 95% CI: 0:82–1.07) and AMHU (Log OR: 0.91; 95% CI: 0.77–1.04). When anti-logged, these estimates show that compared to being a child in RFHU households, the odds of a child becoming stunted were significantly 4.26 times higher in PMHR, 3.25 times higher in PFHR, 2.92 times higher in PMHU, 2.59 times higher in AMHR, and 2.48 times higher in AMHU households. However, RFHR households had the least pooled estimate (Log OR: 0.07; 95% CI: −0.14–0.29), which was not significant, suggesting that PRHR and PFHU were not statistically different (p = 0.51).

Furthermore, the amount of heterogeneity (*I*^*2*^) seen in Figs 4−11 and 14 might not be important (<40%), and the null hypothesis of no heterogeneity is not rejected (p > 0.05). On the other hand, the amount of heterogeneity (*I*^*2*^) observed in Figs 12 and 13 may represent moderate heterogeneity (≤60%); however, the null hypothesis of no heterogeneity is not rejected (p > 0.05).

Table 2 presents the multivariate models showing the adjusted odds ratio for under-five stunting across the 11 household types in comparison to RFHU households. The models acknowledged and adjusted for the effects of Household size, Number of Under-five children in a household, and Age of the Household Head. The models also adjusted for the six clusters in the variable “Region”. Generally, the Model 2 was a better fit than the Model 1 because it had lower deviance. The analysis shows that from 2003 to 2018, five household types, namely PMHR (AOR: 5.12; 3.31, 4.22, & 3.72; p < 0.05), PMHU (AOR: 3.25, 2.75, 2.60, & 2.84; p < 0.05), PFHR (AOR: 3.58, 2.86, 3.47, & 3.07; p < 0.05), AMHU (AOR: 3.96, 2.47, 2.25, & 1.97; p < 0.05), and AMHR (AOR: 4.47, 2.31, 2.37, & 2.08; p < 0.05), consistently had significantly higher odds of predisposing under-five children to stunting than RFHU households.

**Table 2:** Multivariate generalized linear models of under-five stunting by household types, 2003-2018.

Year	2003				2008				2013				2018			
	Model 1		Model 2		Model 1		Model 2		Model 1		Model 2		Model 1		Model 2	
	Unadjusted OR (95% CI)	p-value	Adjusted OR (95% CI)	p-value	Unadjusted OR (95% CI)	p-value	Adjusted OR (95% CI)	p-value	Unadjusted OR (95% CI)	p-value	Adjusted OR (95% CI)	p-value	Unadjusted OR (95% CI)	p-value	Adjusted OR (95% CI)	p-value
Household type*																
RFHU	1.00		1.00		1.00		1.00		1.00		1.00		1.00		1.00	
*PMHR*	5.16 (2.19-12.15)	<0.001	5.12(2.00-13.15)	0.001	3.51(2.43-5.08)	<0.001	3.31(2.28-4.79)	<0.001	4.64(3.05-7.05)	<0.001	4.22(2.65-6.73)	<0.001	4.31 (2.96-6.28)	<0.001	3.72(2.45-5.65)	<0.001
*PMHU*	3.39(1.50-7.64)	0.003	3.25(1.30-8.08)	0.011	2.91(2.15-3.95)	<0.001	2.75(2.08-3.64)	<0.001	2.80(1.12-6.99)	0.028	2.60(1.05-6.40)	0.038	3.06(1.63-5.73)	<0.001	2.84(1.61-5.02)	<0.001
*PFHR*	3.37(1.61-7.05)	0.001	3.58(1.69-7.55)	0.001	2.94(2.25-3.82)	<0.001	2.86(2.13-3.82)	<0.001	3.64(1.98-6.67)	<0.001	3.47(1.90-6.35)	<0.001	3.02(1.622-5.63)	0.001	3.07(1.65-5.72)	<0.001
*PFHU*	4.08(1.17-14.24)	0.027	4.68(1.29-16.96)	0.019	1.45(0.86-2.44)	0.161	1.41(0.81-2.47)	0.223	1.29(0.82-2.04)	0.266	1.30(0.83-2.06)	0.254	1.51(1.13-2.02)	0.006	1.51(1.12-2.02)	0.006
*AMHR*	4.45(1.75-11.31)	0.002	4.47(1.63-12.27)	0.004	2.44(1.85-3.23)	<0.001	2.31(1.70-3.13)	<0.001	2.56(1.53-4.29)	<0.001	2.37(1.36-4.13)	0.002	2.38(1.64-3.46)	<0.001	2.08(1.42-3.03)	<0.001
*AMHU*	3.86(1.45-10.28)	0.007	3.96(1.35-11.59)	0.012	2.59(1.87-3.59)	<0.001	2.47(1.73-3.56)	<0.001	2.46(1.34-4.50)	0.004	2.25(1.21-4.19)	0.011	2.14(1.52-3.03)	<0.001	1.97(1.41-2.77)	<0.001
*AFHR*	3.12(0.92-10.66)	0.069	3.47(0.97-12.44)	0.056	1.88(1.50-2.36)	<0.001	1.86(1.47-2.36)	<0.001	1.50(1.00-2.25)	0.048	1.50(1.00-2.26)	0.051	1.42(1.11-1.81)	0.006	1.48(1.21-1.82)	<0.001
*AFHU*	1.99(0.89-4.50)	0.099	2.31(0.97-5.46)	0.057	1.67(1.13-2.47)	0.010	1.63(1.12-2.39)	0.012	1.80(1.31-2.47)	<0.001	1.77(1.27-2.46)	0.001	1.36(0.88-2.11)	0.167	1.41(0.90-2.21)	0.130
*RMHR*	1.73(0.61-4.92)	0.307	1.71(0.56-5.22)	0.345	1.60(1.25-2.03)	<0.001	1.53(1.18-1.98)	0.001	1.16(0.79-1.71)	0.450	1.09(0.74-1.61)	0.662	1.07(0.74-1.55)	0.709	0.99(0.68-1.42)	0.941
*RMHU*	1.78(0.64-5.00)	0.272	1.83(0.61-5.51)	0.279	1.27(0.82-1.95)	0.287	1.22(0.79-1.89)	0.377	1.33(0.78-2.26)	0.288	1.26(0.74-2.14)	0.386	0.98(0.74-1.29)	0.883	0.94(0.73-1.21)	0.611
*RFHR*	0.57(0.32-1.03)	0.062	0.61(0.36-1.03)	0.063	1.31(1.04-1.65)	0.022	1.29(1.03-1.60)	0.024	0.96(0.75-1.24)	0.770	0.95(0.74-1.22)	0.704	1.05(0.61-1.79)	0.862	1.12(0.70-1.79)	0.637
Number of household members			1.05(1.03-1.07)	<0.001			1.04(1.02-1.06)	<0.001			1.04(1.02-1.07)	0.001			1.08(1.06-1.11)	<0.001
Number of children ≤5 years			1.03(0.97-1.09)	0.349			0.97(0.93-1.02)	0.205			1.00(0.97-1.02)	0.643			0.99(0.94-1.04)	0.652
Age of Household Head			0.99(0.98-1.00)	0.170			0.99(0.99-1.00)	0.001			0.99(0.99-1.00)	0.008			0.99(0.99-0.99)	<0.001
Constant	0.23(0.11-0.49)	<0.001	0.21(0.10-0.45)	<0.001	0.30(0.20-0.45)	<0.001	0.36(0.22-0.60)	<0.001	0.23(0.17-0.30)	<0.001	0.24(0.18-0.34)	<0.001	0.28(0.21-0.36)	<0.001	0.27(0.20-0.37)	<0.001
Deviance	5663.07		5631.63		24617.81		24472.21		30445.66		30352.06		13987.18		13830.54	
Mean VIF			3.26				3.60				2.76				2.65	
N	4,282		4,282		19,010		18,927		24,505		24,505		11,364		11,364	

## Discussion

The prevalence of under-five stunting in Nigeria remains unacceptably high given its debilitating effects and impact on children from childhood to adulthood, and even on the nation. Research shows the cost of stunting, in terms of the reduction in per capita income from some of today’s workforce being stunted in childhood, is between 5–7%, and it takes much longer than 10 years for a childhood stunting rate among workers to fall by 20% [8].

This study applies the concept of intersectionality to describe how systems of nutrition inequality based on the gender of household head, household wealth index, and household residence “intersect” to create unique dynamics and effects that sustained high under-five stunting prevalence in Nigeria from 2003 to 2018 [26]. Although the literature comprises an extensive list of drivers of stunting, the drivers are often presented as independent factors that promote stunting. Since these drivers could be mutually reinforcing, it is necessary that they be analyzed and addressed simultaneously to prevent one driver from reinforcing another. For instance, tackling the household poverty gap alone – without including other dimensions such as the gender of household heads and the type of residence – may likely reinforce stunting among the under-five children. However, there is a dearth of literature looking at the effect or influence of the unique dynamics created by the intersection of these drivers on early childhood stunting, most especially in Africa and Nigeria [23].

From an intersectional lens, the findings of this study suggest that being an under-five child in any of the following households—poor male-headed rural (PMHR) households, poor male-headed urban (PMHU) households, poor female-headed rural (PFHR) households, average male-headed urban (AMHU) households, and average male-headed rural (AMHR) households, had consistently conferred higher odds of being stunted on children aged ≤5years in Nigeria for about 15 years (2003 till 2018) when compared with being an under-five in a rich female-headed urban (RFHU) household. It is interesting to see how household wealth status, household residence, and the gender of household heads reinforced each other to produce the dynamics that had consistently sustained the prevalence of stunting in the country from 2003 to 2018. So, why would the odds of under-five stunting be significantly higher in these five household types? Evidence from previous studies shows that stunting is inversely related to household wealth status [17,18,20], meaning that poor households are at higher odds or risk of stunting. Another previous study conducted in Nigeria demonstrates that poverty is more likely to be entrenched in male-headed households than in female-headed households [27]. Perhaps this partly explains why the odds of stunting were consistently higher in all the poor and the average more male-headed households, irrespective of the place of residence, in this study. On the other hand, the evidence in the literature around the association between place of residence (rural or urban) and stunting is mixed— significant in some studies but not in other studies [17,18,28]. However, in this study, the intersection of household residence with the gender of household head and household wealth status helped differentiate the magnitude of the odds of stunting among households, especially between PFHR and PFHU households. This study shows that children in PFHR households have higher odds of becoming stunted, unlike children in PFHU households, and the only apparent difference between these two households is the location of residence. This finding is somewhat consistent with the findings from some previous studies showing that urbanization is associated with improved child nutritional outcomes in Nigeria [28,29]. A plausible explanation for the difference could be that children in PFHU households could have better or more access to social safety nets that can help prevent under-five stunting than children in PFHR households.

This study importantly shows that, except for PFHR households, poor and average male-headed households, regardless of their place of residence, have significantly higher odds of under-five stunting. This suggests that women in these households may lack the necessary resources, empowerment to care for their under-five children effectively. This observation aligns with previous research that shows that empowering women—especially by increasing their asset ownership and decision-making power—could reduce the likelihood of stunting in children [30]. However, acknowledging the mediating effect of household headship typology is very critical when trying to improve childhood nutrition through women’s empowerment approaches—asset ownership and instrumental agency of women, because the same research shows that children of empowered women in male-headed households were more likely to experience stunting compared to children of mothers from female-headed households [30].

It is undeniable that the persistently high prevalence of under-five stunting has significant policy implications, requiring a comprehensive and coordinated approach. The global implications of these findings indicate that the higher likelihood of under-five stunting in the five specific household types, observed from 2003 to 2018, has likely contributed to Nigeria’s share of the global burden of malnutrition during that period. This situation may have hindered the country’s progress in combating under-five stunting. If current practices continue unchanged, the greater concern is the negative impact that the higher likelihood of under-five stunting in these households may have on achieving the Sustainable Development Goals (SDGs) 1, 2, 3, and 5, as well as ensuring the right to adequate food. The slow rate of decline in under-five stunting prevalence in Nigeria suggests that interventions, including the development and implementation of the National Strategic Plan of Action for Nutrition (2014–2025), effective coordination of stakeholders, and increased government commitment to funding, may not have yielded the desired results based on the available statistics [9]. The high prevalence of under-five stunting persists despite the implementation of nutrition-specific interventions and programs, such as the Bhutta et al. nutrition package, which comprises salt iodization, multiple micronutrient supplementation in pregnancy, including iron-folate, calcium supplementation in pregnancy, energy-protein supplementation in pregnancy, vitamin A supplementation in childhood, zinc supplementation in childhood, breastfeeding promotion, complementary feeding education, complementary food supplementation, and severe acute malnutrition management [31].

The findings of this study, however, offer a crucial advancement by pinpointing vulnerable households that may benefit from nutrition-sensitive interventions aimed at preventing under-five stunting. To effectively address this issue, policymakers and program planners are expected to tailor and prioritize their strategies based on empirical evidence [32]. This study identifies five high-risk households that may require more nutrition-specific interventions, such as improving perinatal and maternal conditions, as well as enhancing feeding practices, including breastfeeding, which could contribute to the elevated risk of under-five stunting [16]; and more nutrition-sensitive interventions (such as Agriculture and Food security, Social Protection, Early Childhood Development and Education, Maternal Mental Health, Women’s Empowerment, Child protection, Water and Sanitation, Health and Family Planning services, Poverty Alleviation, and Schooling) that aim to address the more immediate causes of undernutrition, such as inadequate dietary intake and poor health [33,34]. Research has shown that the unique sets of stunting determinants that predicted stunting reduction within countries that have reduced stunting include improvements in maternal and paternal education, household socioeconomic status, sanitation conditions, maternal health services access, and family planning [35]. To achieve Nigeria’s goal of reducing stunting among children under five by 40% by 2025, it is essential to significantly increase the coverage of stunting-prevention activities to 90%, with particular emphasis on these high-risk households [8,36]. The country may also need to make some strategic adjustments in alignment with the initiatives of the WHO Department of Maternal, Newborn, Child, and Adolescent Health and Ageing to effectively address challenges and leverage existing opportunities. For example, it should respond to demographic changes and emerging threats, including the impacts of climate change and increasing urbanization on the health and well-being of women, newborns, children, and adolescents. Additionally, there should be a greater focus on humanitarian efforts and support in fragile settings to ensure that no one is left behind [32].

The key strengths of this study include the large datasets used for the analysis and the generalizability of the study findings because the surveys were nationally representative. It also includes the use of an objective measurement of the outcome variable (under-five stunting). However, this study has a few limitations. First, the study datasets, being quantitative, could not provide insights into why some households consistently have higher odds of under-five stunting than others. Additionally, there were some variables that were not included in this study because of the study’s objectives and data availability. The variables included factors related to the index child and the mother, and environmental determinants of health. So, omitted variable residuals might exist because the models did not include those variables, and the effects of those unaccounted factors might have been absorbed into the residuals, which might make the residuals not really pure random noise.

## Conclusions

The prevalence of stunting among children under five in Nigeria remains unacceptably high. Despite numerous interventions and investments over the years, the reduction in prevalence from 46% in 1999 to just 37% in 2018 indicates limited progress. The long-term effects of this chronic nutritional issue on Nigeria’s human capital and its economic development may be severe and far-reaching. If the country does not adopt new strategies to reduce stunting from its current persistent double-digit rate to a single digit, its future is at risk. The findings of this study, therefore, offer opportunities for further research, besides providing fresh insights that could guide policymakers in developing new policies and help program managers design and implement more effective nutrition-sensitive interventions and programs, which should address the underlying and basic causes of undernutrition (e.g., poverty, food insecurity, education, women’s empowerment, and social status) in households most at risk of under-five stunting through indirect but plausible pathways [33]. There is a need for a thorough analysis of other interacting factors; without it, the development of public policies risks being both limited and suboptimal, as current literature indicates.

## Supporting information

S1 File Weighted stunting prevelance dataset. (XLSX)

S2 File Forestplots datasets. (XLSX)

S3 File Data management and analysis. (DO)

S4 File The final Study dataset. (BZ2)
