# Supplementary material for: Intersectional analysis of early childhood stunting in households in Nigeria
Source: PLoS One. 2026 Aug 20;21(8):e0355408. doi: 10.1371/journal.pone.0355408 (PMC13492823; doi:10.1371/journal.pone.0355408)
Supplement: Peer review history

# Peer review: Decision Letter 0

**DOI:** 10.1371/journal.pone.0355408.r001

**Authors:** Ashish Khobragade

6 Jul 2025

Dear Dr. Oginni,

Thank you for submitting your manuscript to PLOS ONE. After careful consideration, we feel that it has merit but does not fully meet PLOS ONE’s publication criteria as it currently stands. Therefore, we invite you to submit a revised version of the manuscript that addresses the points raised during the review process.

Please submit your revised manuscript by  Aug 20 2025 11:59PM. If you will need more time than this to complete your revisions, please reply to this message or contact the journal office at plosone@plos.org. When you're ready to submit your revision, log on to https://www.editorialmanager.com/pone/ and select the 'Submissions Needing Revision' folder to locate your manuscript file.

- A rebuttal letter that responds to each point raised by the academic editor and reviewer(s). You should upload this letter as a separate file labeled 'Response to Reviewers'.
- A marked-up copy of your manuscript that highlights changes made to the original version. You should upload this as a separate file labeled 'Revised Manuscript with Track Changes'.
- An unmarked version of your revised paper without tracked changes. You should upload this as a separate file labeled 'Manuscript'.

If you would like to make changes to your financial disclosure, please include your updated statement in your cover letter. Guidelines for resubmitting your figure files are available below the reviewer comments at the end of this letter.

If applicable, we recommend that you deposit your laboratory protocols in protocols.io to enhance the reproducibility of your results. Protocols.io assigns your protocol its own identifier (DOI) so that it can be cited independently in the future. For instructions see: https://journals.plos.org/plosone/s/submission-guidelines#loc-laboratory-protocols. Additionally, PLOS ONE offers an option for publishing peer-reviewed Lab Protocol articles, which describe protocols hosted on protocols.io. Read more information on sharing protocols at https://plos.org/protocols?utm_medium=editorial-email&utm_source=authorletters&utm_campaign=protocols.

We look forward to receiving your revised manuscript.

Kind regards,

Ashish Wasudeo Khobragade, MD

Academic Editor

PLOS ONE

Journal Requirements:

When submitting your revision, we need you to address these additional requirements.

1. Please ensure that your manuscript meets PLOS ONE's style requirements, including those for file naming. The PLOS ONE style templates can be found at

https://journals.plos.org/plosone/s/file?id=wjVg/PLOSOne_formatting_sample_main_body.pdf and

https://journals.plos.org/plosone/s/file?id=ba62/PLOSOne_formatting_sample_title_authors_affiliations.pdf

2. When completing the data availability statement of the submission form, you indicated that you will make your data available on acceptance. We strongly recommend all authors decide on a data sharing plan before acceptance, as the process can be lengthy and hold up publication timelines. Please note that, though access restrictions are acceptable now, your entire data will need to be made freely accessible if your manuscript is accepted for publication. This policy applies to all data except where public deposition would breach compliance with the protocol approved by your research ethics board. If you are unable to adhere to our open data policy, please kindly revise your statement to explain your reasoning and we will seek the editor's input on an exemption. Please be assured that, once you have provided your new statement, the assessment of your exemption will not hold up the peer review process.

Additional Editor Comments:

1. A multivariate generalized linear model is presented in Table 2. How many dependent variables are analyzed? Which ‘link’ function is used? Mention the results of the Goodness of fit test for the model.

2. As the analysis is based on secondary data, mention the residuals that were not considered in the model in the limitations section. How may this affect the findings?

3. In Table 2, some p-values are mentioned as 0.000; mention them as <0.001.

[Note: HTML markup is below. Please do not edit.]

Reviewers' comments:

Reviewer's Responses to Questions

**Comments to the Author**

1. Is the manuscript technically sound, and do the data support the conclusions?

Reviewer #1: Yes

Reviewer #2: Partly

Reviewer #3: Yes

**********

2. Has the statistical analysis been performed appropriately and rigorously?

Reviewer #1: Yes

Reviewer #2: No

Reviewer #3: Yes

**********

3. Have the authors made all data underlying the findings in their manuscript fully available?

The PLOS Data policy

Reviewer #1: Yes

Reviewer #2: No

Reviewer #3: Yes

**********

4. Is the manuscript presented in an intelligible fashion and written in standard English?

Reviewer #1: Yes

Reviewer #2: Yes

Reviewer #3: Yes

**********

Reviewer #1: Thank you for the opportunity to review this paper. It was well done and was a very interesting read. My only request is to add additional information about the groups in the methods section. Specifically stating how it was determined that a home was male vs female headed, as well as the income requirements for each group.

Reviewer #2: Abstract: Please define all abbreviations upon first use to ensure clarity for a broad readership.

Literature: The author should clarify why the 2023/24 report has not been incorporated into the analysis, given its relevance and recency.

Survey Design: The analysis does not appear to account for the stratification, clustering, and weighting inherent in the complex DHS survey design. Please explain this omission or adjust the analysis accordingly.

Reproducibility: For transparency and reproducibility, I recommend the author upload the complete Stata code (including both data recoding and table generation) to a public repository such as GitHub.

Analytical Approach: The rationale for employing a meta-analysis across survey rounds instead of conducting a pooled analysis is unclear, especially given the standardized nature of DHS data across years. Please justify this methodological choice.

Covariate Selection: The basis for selecting the included covariates needs to be elaborated. Were these variables chosen based on theory, prior research, or data availability?

Multicollinearity: It is important to assess multicollinearity among covariates. Please indicate whether this was done and report the variance inflation factor (VIF) values, if available.

Confounding: There is a significant risk of residual confounding. The author should explain the criteria used for selecting confounders and discuss potential unmeasured confounders.

Reviewer #3: The article presents a well-constructed analysis of the statistical model selection, sample, and variables included. However, it neglects to address critical variables that significantly impact these outcomes. Environmental determinants of health intersect with nutritional outcomes, such as stunted growth. Although the primary aim is not to examine these factors in detail, it is pertinent to at least reference them within the background and discussion sections, as the current scope appears constrained in light of contemporary scientific evidence. It is recommended to incorporate a review of the document "The State of Food Security and Nutrition in the World 2024"https://www.fao.org/publications/fao-flagship-publications/the-state-of-food-security-and-nutrition-in-the-world/2021/en, for additional context.

Furthermore, it is advisable to clearly define the analytical baseline, particularly if comparisons are drawn with international statistics. If such comparisons are made, explicit identification of the reference datasets and their temporal context is necessary. While the assertions made are generally accurate, they lack grounding due to the absence of explicitly stated reference standards.

Lastly, the discussion section could benefit from a more in-depth exploration of the global implications of the observed high prevalence rates, especially concerning their impact on achieving Sustainable Development Goals (SDGs) 1, 2, 3, and 5, as well as the right to adequate food. It is particularly relevant as the conclusion emphasizes the need for interventions to address the identified variables. Without a thorough analysis of the interacting factors, the development of public policies risks being both limited and suboptimal, as indicated by current literature. It is suggested to review the document "Department of Maternal, Newborn, Child and Adolescent Health and Ageing: Strategic Directions 2023-2030", to strengthen the analysis further. https://www.who.int/publications/i/item/9789240100657

**********

what does this mean?). If published, this will include your full peer review and any attached files.

If you choose “no”, your identity will remain anonymous but your review may still be made public.

**Do you want your identity to be public for this peer review?** For information about this choice, including consent withdrawal, please see our Privacy Policy

Reviewer #1: No

Reviewer #2: No

Reviewer #3: **Yes:** Alondra Coral Aragon-Gama

**********

[NOTE: If reviewer comments were submitted as an attachment file, they will be attached to this email and accessible via the submission site. Please log into your account, locate the manuscript record, and check for the action link "View Attachments". If this link does not appear, there are no attachment files.]

While revising your submission, please upload your figure files to the Preflight Analysis and Conversion Engine (PACE) digital diagnostic tool, https://pacev2.apexcovantage.com/. PACE helps ensure that figures meet PLOS requirements. To use PACE, you must first register as a user. Registration is free. Then, login and navigate to the UPLOAD tab, where you will find detailed instructions on how to use the tool. If you encounter any issues or have any questions when using PACE, please email PLOS at figures@plos.org. Please note that Supporting Information files do not need this step.

# Author comment: Author response to Decision Letter 1

**DOI:** 10.1371/journal.pone.0355408.r002

12 Oct 2025

Additional Editor Comments:

1. A multivariate generalized linear model is presented in Table 2. How many dependent variables are analyzed? Which ‘link’ function is used? Mention the results of the goodness-of-fit test for the model.

2. As the analysis is based on secondary data, mention the residuals that were not considered in the model in the limitations section. How may this affect the findings?

3. In Table 2, some p-values are mentioned as 0.000; mention them as <0.001.

Author’s Response: Only one dependent variable, “Stunting (Under-five),” was analyzed. Here is the link function used for the model

Model 1: ln⁡〖(Odds(y=1))〗=α+β_1 x

Model 2: ln⁡〖(Odds(y=1))〗=α+β_1 x+β_2 x_2+β_2 x_2+β_3 x_3

The goodness-of-fit test statistics (Deviance) comparing the fitted models to the saturated model (perfect fit) are now reported in the revised manuscript (Table 2).

Some variables were excluded from this study due to the research objectives and data availability. These excluded variables included factors related to the index child, the mother, and environmental determinants of health. As a result, there may be omitted variable residuals because the models did not account for these factors. The effects of these unaccounted variables could have influenced the residuals, leading them to not be purely random noise. This limitation is acknowledged in the revised manuscript.

The p-values mentioned as 0.000 have been changed to <0.001.

Reviewer's Responses to Questions

Comments to the Author

1. Is the manuscript technically sound, and do the data support the conclusions?

The manuscript must describe a technically sound piece of scientific research with data that supports the conclusions. Experiments must have been conducted rigorously, with appropriate controls, replication, and sample sizes. The conclusions must be drawn appropriately based on the data presented.

Reviewer #1: Yes

Reviewer #2: Partly

Reviewer #3: Yes

________________________________________

2. Has the statistical analysis been performed appropriately and rigorously?

Reviewer #1: Yes

Reviewer #2: No

Reviewer #3: Yes

________________________________________

3. Have the authors made all data underlying the findings in their manuscript fully available?

The PLOS Data policy requires authors to make all data underlying the findings described in their manuscript fully available without restriction, with rare exception (please refer to the Data Availability Statement in the manuscript PDF file). The data should be provided as part of the manuscript or its supporting information, or deposited to a public repository. For example, in addition to summary statistics, the data points behind means, medians and variance measures should be available. If there are restrictions on publicly sharing data—e.g. participant privacy or use of data from a third party—those must be specified.

Reviewer #1: Yes

Reviewer #2: No

Reviewer #3: Yes

________________________________________

4. Is the manuscript presented in an intelligible fashion and written in standard English?

PLOS ONE does not copyedit accepted manuscripts, so the language in submitted articles must be clear, correct, and unambiguous. Any typographical or grammatical errors should be corrected at revision, so please note any specific errors here.

Reviewer #1: Yes

Reviewer #2: Yes

Reviewer #3: Yes

________________________________________

5. Review Comments to the Author

Please use the space provided to explain your answers to the questions above. You may also include additional comments for the author, including concerns about dual publication, research ethics, or publication ethics. (Please upload your review as an attachment if it exceeds 20,000 characters)

Reviewer #1: Thank you for the opportunity to review this paper. It was well done and was a very interesting read. My only request is to add additional information about the groups in the methods section. Specifically stating how it was determined that a home was male vs female-headed, as well as the income requirements for each group.

Author’s Response: This has been added in the revised manuscript.

“The three variables used to create the main independent variable were “type of place of residence” (v102: 1-Urban, 2-Rural), “sex of household head” (v151: 1-male, 2-female), and “recoded wealth index (v190)”. The original variable “wealth index (v190)” has 6 categories (1-Poorest, 2-Poor, 3-Middle, 5-Rich, and 6-Richest), but it was recoded into a new variable with three categories (1-Poor, 2-Average, and 3-Rich). Combining the categories of these three variables gave 12 possible combinations, as shown in Table 1.”

Reviewer #2: Abstract: Please define all abbreviations upon first use to ensure clarity for a broad readership.

Author’s Response: I understand your concern, but I could only define the abbreviation for the RFHU due to the abstract's limited word count. Including definitions for all abbreviations would cause the abstract to exceed this limit.

Literature: The author should clarify why the 2023/24 report has not been incorporated into the analysis, given its relevance and recency.

Author’s Response: At the time this study was conducted, the data for 2023/24 were not available.

Survey Design: The analysis does not appear to account for the stratification, clustering, and weighting inherent in the complex DHS survey design. Please explain this omission or adjust the analysis accordingly.

Author’s Response: The analyses accounted for clustering and weighting. Weighted data were used for the analysis. The models also accounted for the clustering effect of the variable “Region” having six clusters, and used the clustered robust standard error estimator.

Reproducibility: For transparency and reproducibility, I recommend the author upload the complete Stata code (including both data recoding and table generation) to a public repository such as GitHub.

Author’s Response: The Stata code file and the study datasets are uploaded

Analytical Approach: The rationale for employing a meta-analysis across survey rounds instead of conducting a pooled analysis is unclear, especially given the standardized nature of DHS data across years. Please justify this methodological choice.

Author’s Response: Although the DHS data are standardized across survey rounds, I employed forest plot meta-analysis rather than a pooled analysis to account for potential heterogeneity across survey years. Each survey round represents an independent population-based sample conducted at different time points, often under varying contextual, policy, and programmatic environments that may influence the associations under study. The forest plot meta-analytic approach allowed me to estimate effect sizes within each survey independently, preserving within-survey variance; quantify between-survey heterogeneity using statistics such as I²; and obtain an overall summary estimate that reflects both within- and between-survey variability. This method provides a more conservative and transparent synthesis of evidence over time than a pooled analysis, which assumes homogeneity and a common error structure across all survey rounds.

Covariate Selection: The basis for selecting the included covariates needs to be elaborated. Were these variables chosen based on theory, prior research, or data availability?

Author’s Response: The selection of covariates was guided by the theoretical framework and the objectives of the study, which focused exclusively on household-level variables. However, the limited number of covariates included in the analysis was primarily due to data availability. More covariates could have been considered if additional variables had been available in the dataset.

Multicollinearity: It is important to assess multicollinearity among covariates. Please indicate whether this was done and report the variance inflation factor (VIF) values, if available.

Author’s Response: It is done and reported in the revised manuscript.

Confounding: There is a significant risk of residual confounding. The author should explain the criteria used for selecting confounders and discuss potential unmeasured confounders.

Author’s Response: I recognized that residual confounding is a significant limitation of the study. The selection of confounders or control variables was determined based on the study's objectives and the availability of data. The study aims to focus solely on household-level variables. In the revised manuscript, I have addressed the potential impact of unmeasured confounders.

Reviewer #3: The article presents a well-constructed analysis of the statistical model selection, sample, and variables included. However, it neglects to address critical variables that significantly impact these outcomes. Environmental determinants of health intersect with nutritional outcomes, such as stunted growth. Although the primary aim is not to examine these factors in detail, it is pertinent to at least reference them within the background and discussion sections, as the current scope appears constrained in light of contemporary scientific evidence. It is recommended to incorporate a review of the document "The State of Food Security and Nutrition in the World 2024"https://www.fao.org/publications/fao-flagship-publications/the-state-of-food-security-and-nutrition-in-the-world/2021/en, for additional context.

Furthermore, it is advisable to clearly define the analytical baseline, particularly if comparisons are drawn with international statistics. If such comparisons are made, explicit identification of the reference datasets and their temporal context is necessary. While the assertions made are generally accurate, they lack grounding due to the absence of explicitly stated reference standards.

Lastly, the discussion section could benefit from a more in-depth exploration of the global implications of the observed high prevalence rates, especially concerning their impact on achieving Sustainable Development Goals (SDGs) 1, 2, 3, and 5, as well as the right to adequate food. It is particularly relevant as the conclusion emphasizes the need for interventions to address the identified variables. Without a thorough analysis of the interacting factors, the development of public policies risks being both limited and suboptimal, as indicated by current literature. It is suggested to review the document "Department of Maternal, Newborn, Child and Adolescent Health and Ageing: Strategic Directions 2023-2030", to strengthen the analysis further. https://www.who.int/publications/i/item/9789240100657

Author’s Response: The revised manuscript has a few critical variables, including the environmental determinants of health within the background and discussion sections, and incorporates the suggested literature.

________________________________________

6. PLOS authors have the option to publish the peer review history of their article (what does this mean?). If published, this will include your full peer review and any attached files.

If you choose “no”, your identity will remain anonymous but your review may still be made public.

Do you want your identity to be public for this peer review? For information about this choice, including consent withdrawal, please see our Privacy Policy.

Reviewer #1: No

Reviewer #2: No

Reviewer #3: Yes: Alondra Coral Aragon-Gama

________________________________________

Attachment Submitted filename: Response to Reviewers comments.docx

# Peer review: Decision Letter 1

**DOI:** 10.1371/journal.pone.0355408.r003

**Authors:** Ashish Khobragade

21 Jul 2026

Intersectional analysis of early childhood stunting in households in Nigeria

PONE-D-25-09389R1

Dear Dr. Oginni,

We’re pleased to inform you that your manuscript has been judged scientifically suitable for publication and will be formally accepted for publication once it meets all outstanding technical requirements.

Within one week, you’ll receive an e-mail detailing the required amendments. When these have been addressed, you’ll receive a formal acceptance letter and your manuscript will be scheduled for publication.

An invoice will be generated when your article is formally accepted. Please note, if your institution has a publishing partnership with PLOS and your article meets the relevant criteria, all or part of your publication costs will be covered. Please make sure your user information is up-to-date by logging into Editorial Manager at Editorial Manager® and clicking the ‘Update My Information' link at the top of the page. For questions related to billing, please contact billing support.

If your institution or institutions have a press office, please notify them about your upcoming paper to help maximize its impact. If they’ll be preparing press materials, please inform our press team as soon as possible -- no later than 48 hours after receiving the formal acceptance. Your manuscript will remain under strict press embargo until 2 pm Eastern Time on the date of publication. For more information, please contact onepress@plos.org.

Kind regards,

Ashish Wasudeo Khobragade, MD

Academic Editor

PLOS One

Additional Editor Comments (optional):

Reviewers' comments:

Reviewer's Responses to Questions

**Comments to the Author**

Reviewer #3: All comments have been addressed

**********

2. Is the manuscript technically sound, and do the data support the conclusions?

Reviewer #3: Yes

**********

3. Has the statistical analysis been performed appropriately and rigorously?

Reviewer #3: I Don't Know

**********

4. Have the authors made all data underlying the findings in their manuscript fully available?

The PLOS Data policy

Reviewer #3: Yes

**********

5. Is the manuscript presented in an intelligible fashion and written in standard English?

Reviewer #3: Yes

**********

Reviewer #3: The document is ready to be publish. Thanks for including our suggestions...........................

**********

what does this mean?). If published, this will include your full peer review and any attached files.

If you choose “no”, your identity will remain anonymous but your review may still be made public.

**Do you want your identity to be public for this peer review?**  For information about this choice, including consent withdrawal, please see our Privacy Policy

Reviewer #3: **Yes:** Alondra Coral Aragon Gama

**********

# Editor report: Acceptance letter

**DOI:** 10.1371/journal.pone.0355408.r004

**Authors:** Ashish Khobragade

PONE-D-25-09389R1

PLOS One

Dear Dr. Oginni,

I'm pleased to inform you that your manuscript has been deemed suitable for publication in PLOS One. Congratulations! Your manuscript is now being handed over to our production team.

At this stage, our production department will prepare your paper for publication. This includes ensuring the following:

* All references, tables, and figures are properly cited

* All relevant supporting information is included in the manuscript submission,

* There are no issues that prevent the paper from being properly typeset

You will receive further instructions from the production team, including instructions on how to review your proof when it is ready. Please keep in mind that we are working through a large volume of accepted articles, so please give us a few days to review your paper and let you know the next and final steps.

Lastly, if your institution or institutions have a press office, please let them know about your upcoming paper now to help maximize its impact. If they'll be preparing press materials, please inform our press team within the next 48 hours. Your manuscript will remain under strict press embargo until 2 pm Eastern Time on the date of publication. For more information, please contact onepress@plos.org.

You will receive an invoice from PLOS for your publication fee after your manuscript has reached the completed accept phase. If you receive an email requesting payment before acceptance or for any other service, this may be a phishing scheme. Learn how to identify phishing emails and protect your accounts at https://explore.plos.org/phishing.

If we can help with anything else, please email us at customercare@plos.org.

Thank you for submitting your work to PLOS ONE and supporting open access.

Kind regards,

PLOS ONE Editorial Office Staff

on behalf of

Dr. Ashish Wasudeo Khobragade

Academic Editor

PLOS One
